# Supplementary material for: Peptide binder design with inverse folding and protein structure prediction
Source: Commun Chem. 2023 Oct 25;6:229. doi: 10.1038/s42004-023-01029-7 (PMC10600234; doi:10.1038/s42004-023-01029-7)
Supplement: Supplementary file 2 — Supplementary information [file 42004_2023_1029_MOESM2_ESM.pdf]

Supplementary material for

Peptide binder design with inverse folding and protein structure prediction

Patrick Bryant[1,2] and Arne Elofsson[1,2]

[1]Science for Life Laboratory, 172 21 Solna, Sweden
[2]Department of Biochemistry and Biophysics, Stockholm University, 106 91 Stockholm, Sweden
Email: arne@bioinfo.se

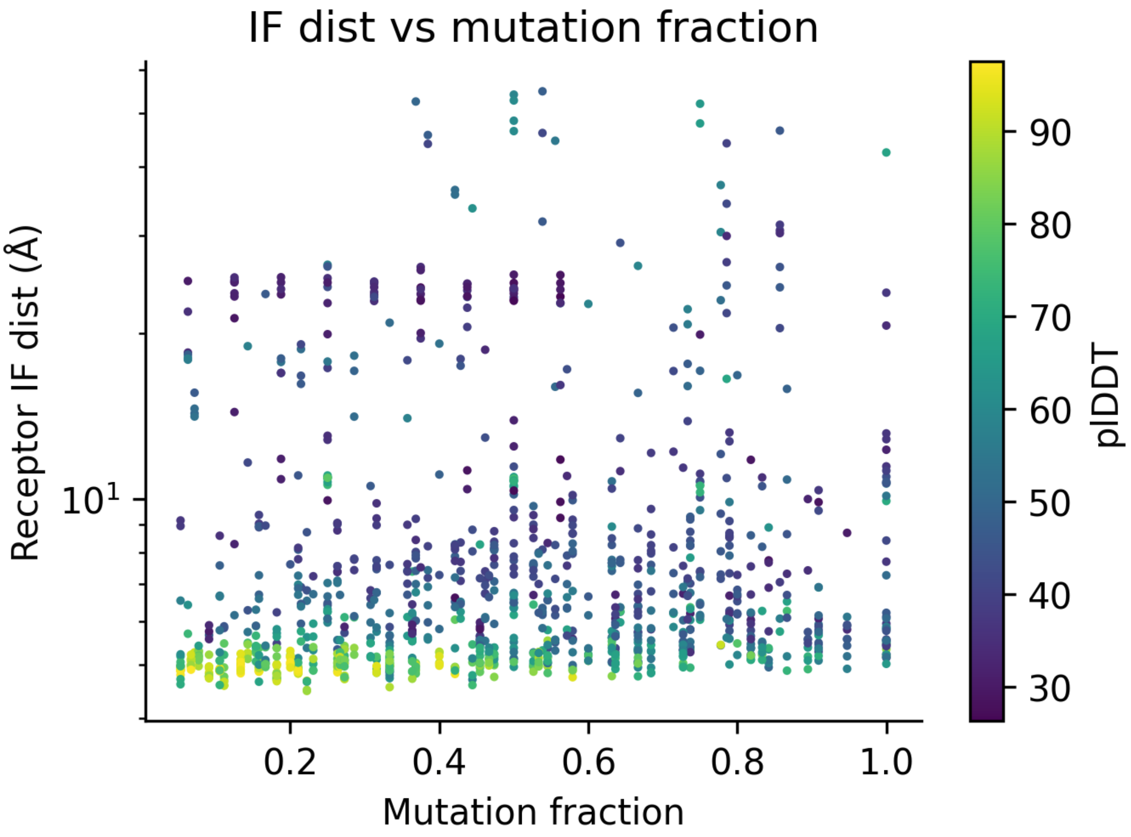

Supplementary Figure 1. Relationship between the mutation fraction and distance to the receptor interface for the 12 peptides that could be predicted using AF at <2Å RMSD. Each sample (n=1160) is colored by plDDT. The y-axis starts at 4 Å and is displayed in logarithmic scale.

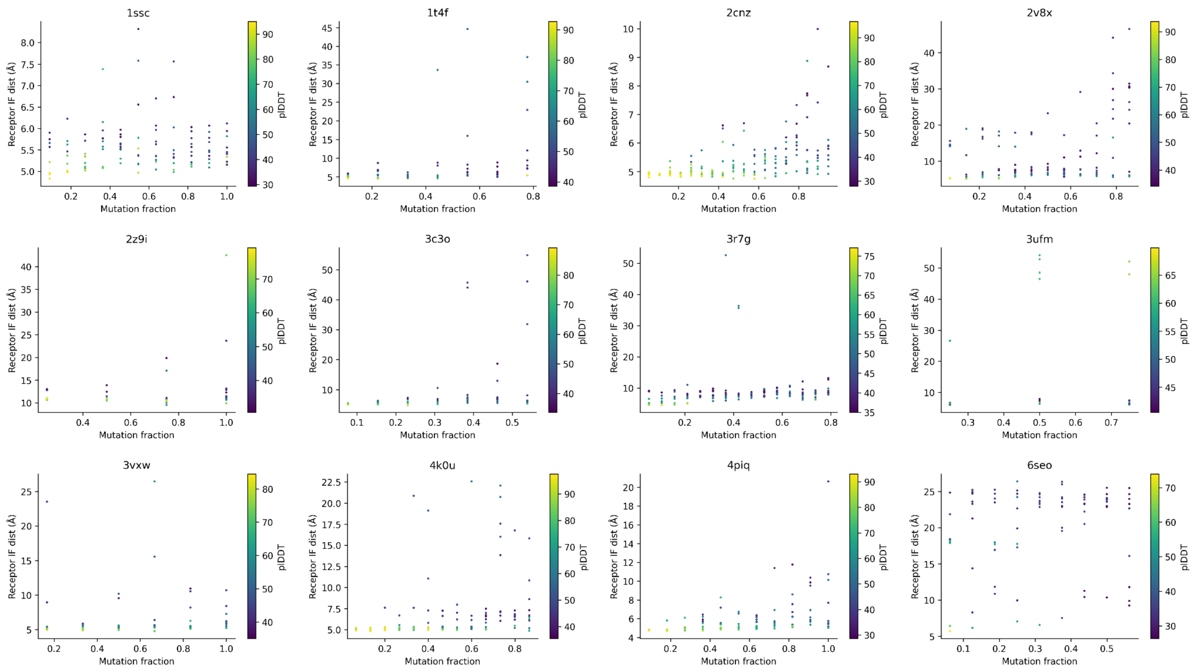

Supplementary Figure 2. Relationship between the mutation fraction and distance to the receptor interface for each of the 12 peptides that could be predicted using AF at <2Å RMSD. Each sample is colored by plDDT.

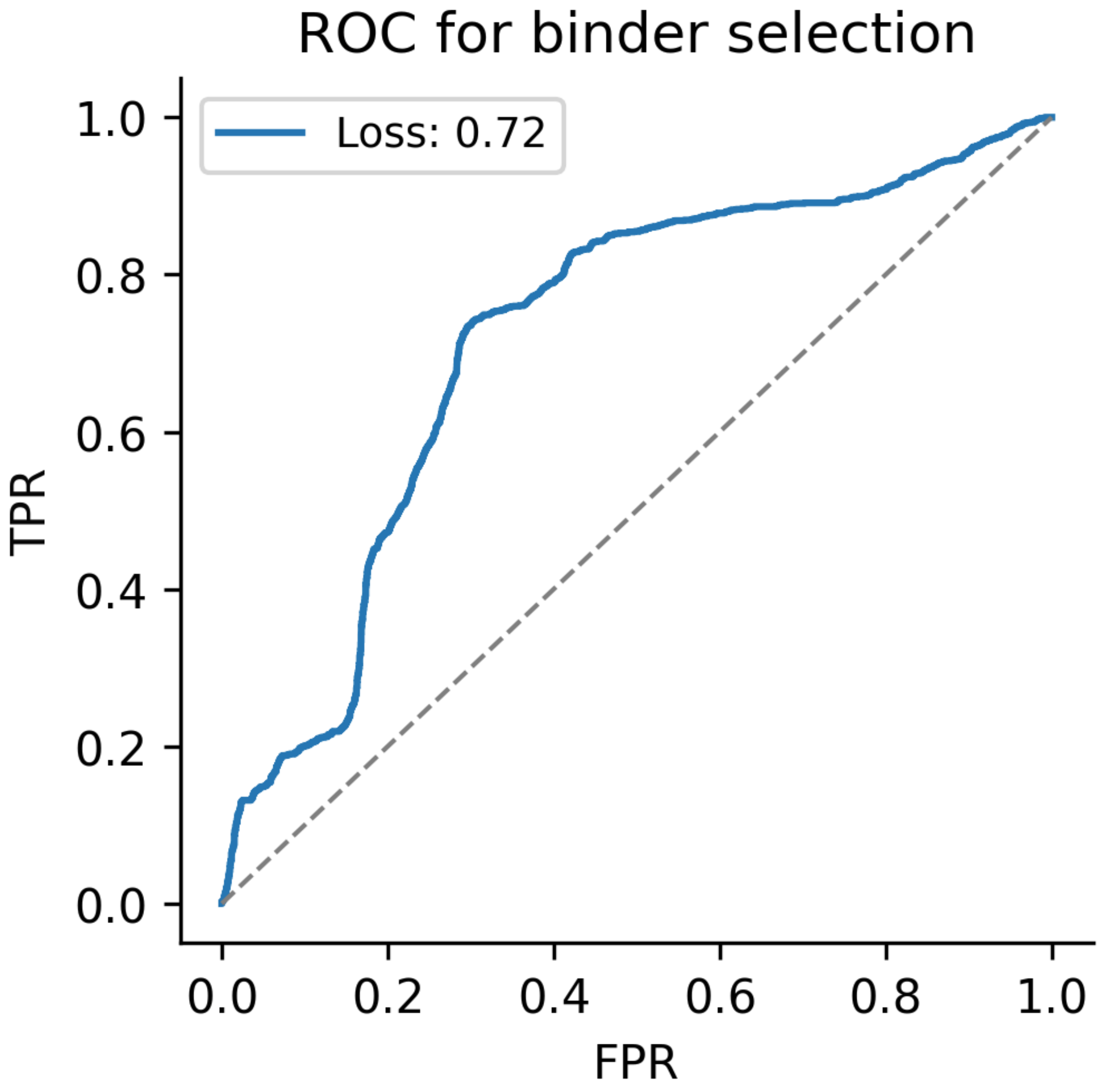

Supplementary Figure 3. ROC curve for selecting miniprotein binders with Normalised NGS counts above 0.01 using the loss as a thresholding function. The NGS counts were normalised by dividing with the highest observed count. The AUC is 0.72, resulting in 20% of the binders can be selected at an FPR of 10%.

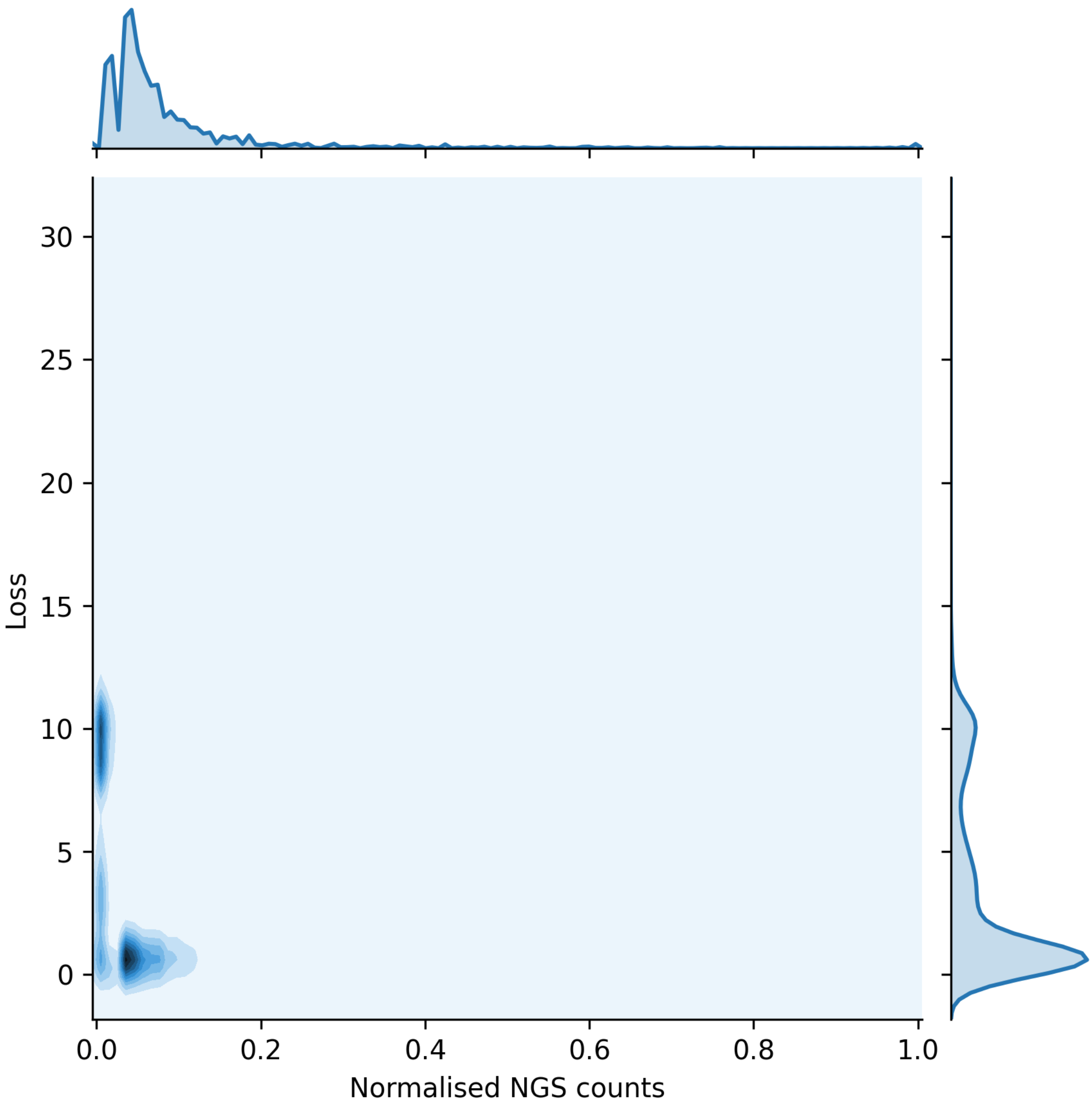

Supplementary Figure 4. Normalised NGS counts and loss. The NGS counts were normalised by dividing with the highest observed count. There is a stark difference between the loss at zero counts and counts above zero.

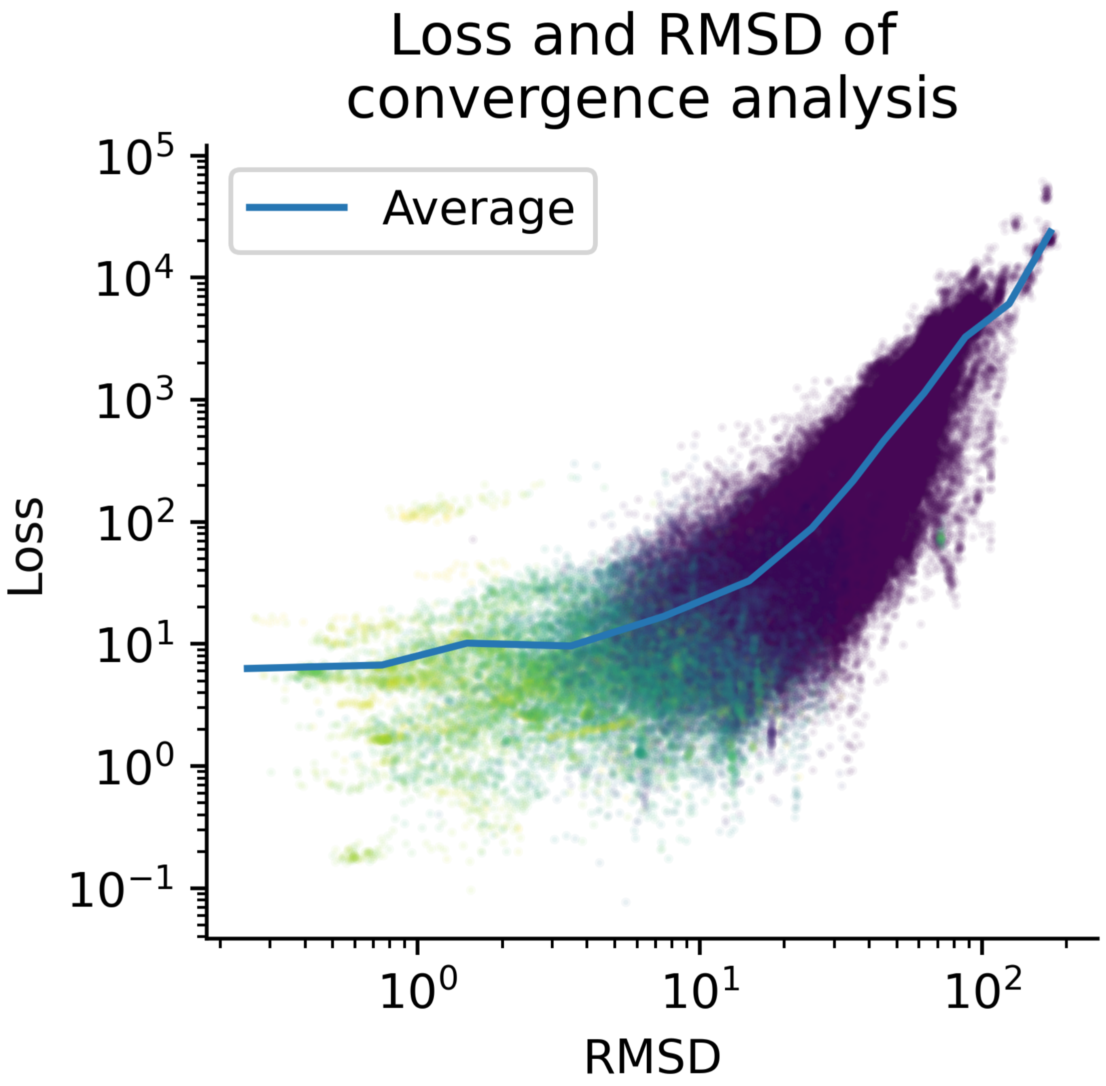

Supplementary Figure 5. Loss and RMSD colored by the fraction of recovered contacts for all designs in the convergence analysis (n=282853). The colormap is the same as in Figure 4a (yellow=1, purple=0).

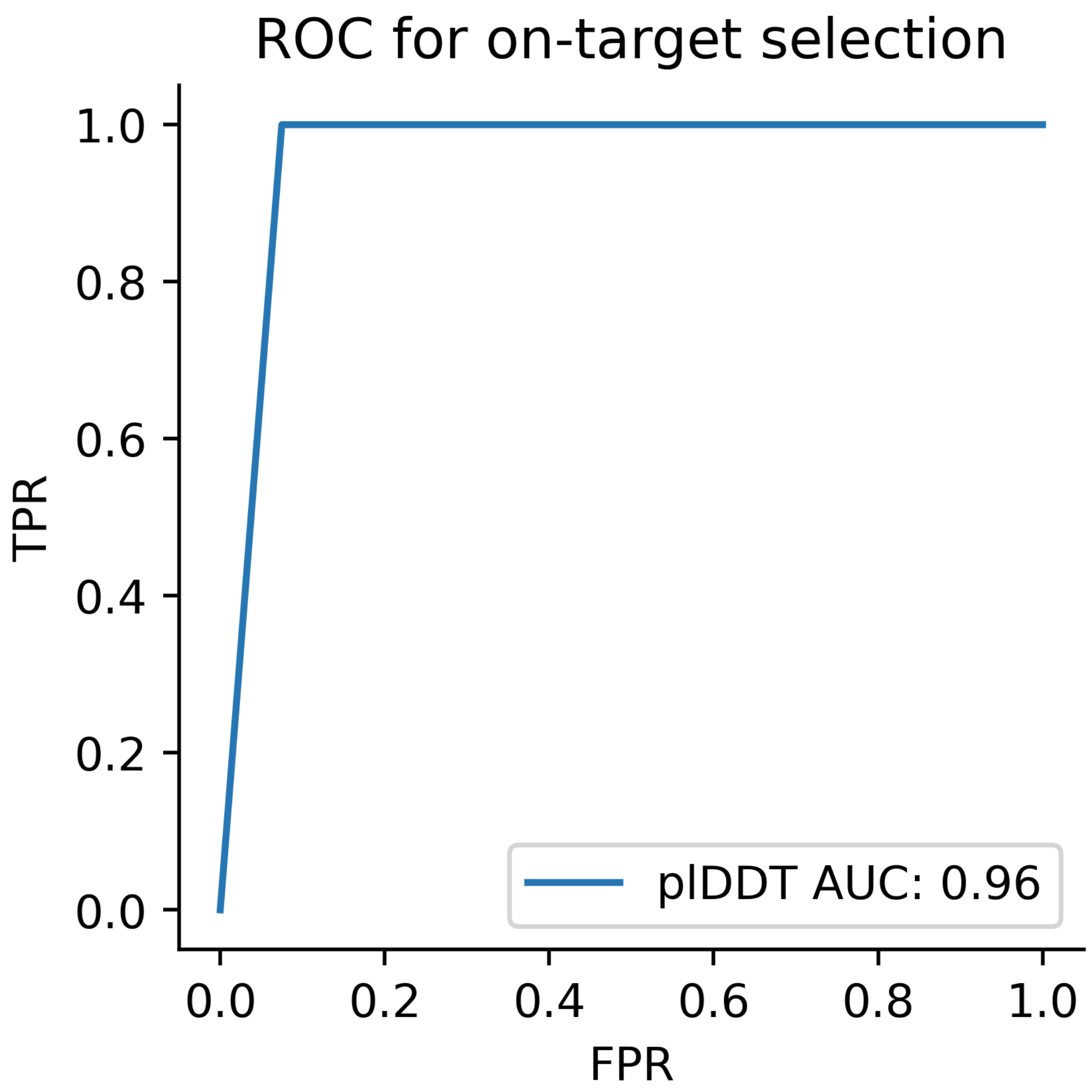

Supplementary Figure 6. TPR vs FPR for selecting designed binders towards intended target structures for 185 successful binder designs compared to 100 randomly selected target proteins (n=18500). Using the loss function, the ROC AUC=0.96. At an FPR of 10%, 100% of successful designs can be selected using higher plDDT as the discriminator.

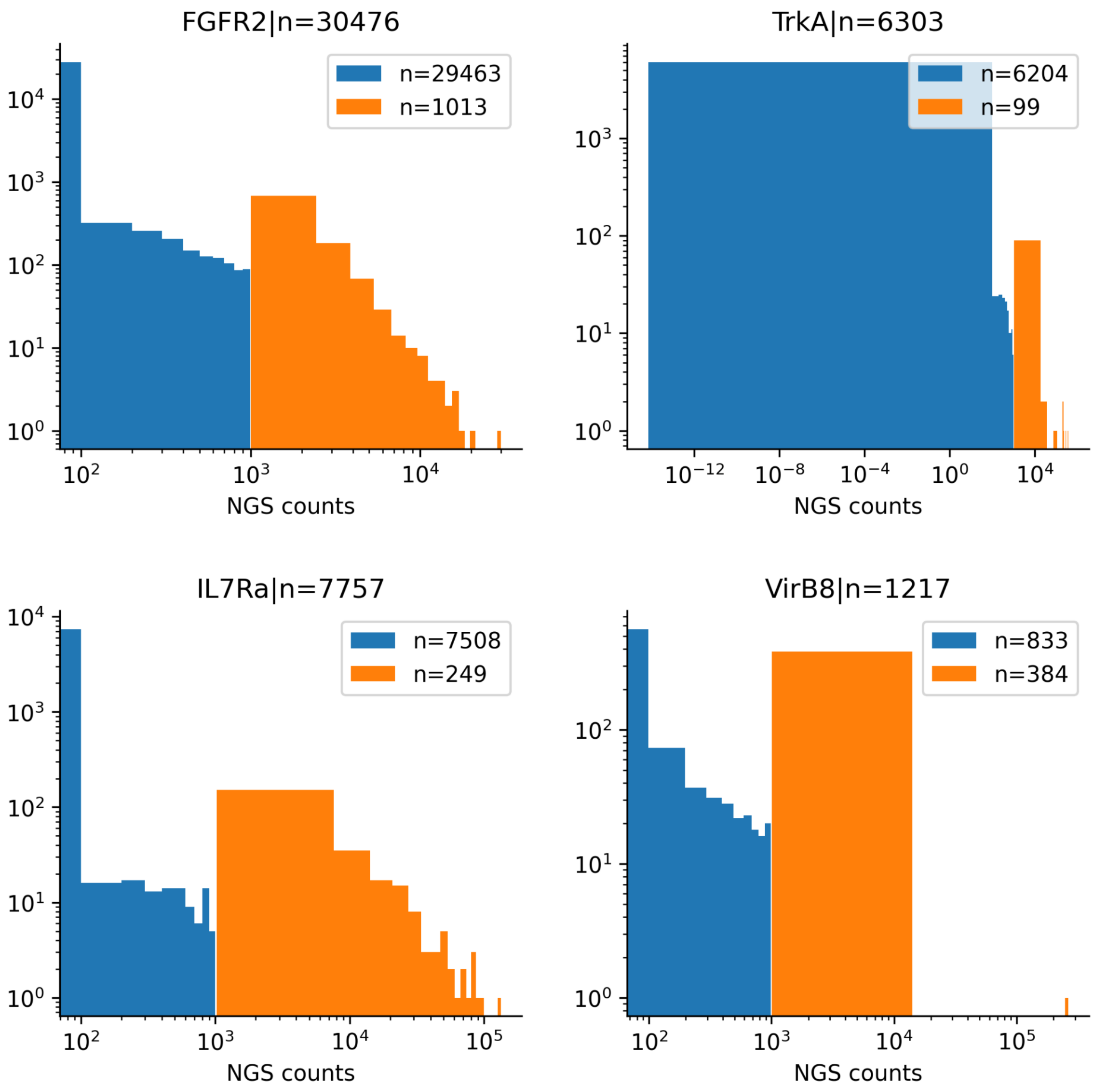

Supplementary Figure 7. NGS counts for the four analysed miniprotein binders. The blue histograms represent the NGS counts below 1000 and the orange those of ≥1000.

Supplementary Table 1. The number of successful runs out of the number of possible ones for the different analyses performed throughout the study.

Task	Number of possible runs	Number of successful runs
Zero-shot prediction, seed length 10	2926	2895
Zero-shot prediction, seed length 20	2926	2805
Zero-shot prediction, seed length 30	2926	2672

Zero-shot prediction, seed length 40	2926	2451
Zero-shot prediction, seed length 50	2926	2333
Binder design convergence, 100 runs for seed length 10	2926 targets (292’600 samples)	2843 targets (282’853 samples)
Specificity analysis (off-target effects) for the 185 successful designs vs 100 randomly selected proteins	18500	18500
Protein MPNN homomers using ESM-IF1	1172 targets (117’200 samples)	1172 targets (115’855 samples)
Protein MPNN homomers using ProteinMPNN	1172 targets (117’200 samples)	1172 targets (116’334 samples)

Supplementary notes

Regarding the analysis of the designed minibinders in Figure 2, we have analysed the false positive rate (FPR) along with the inflated plDDTs (compared to the peptide binders in Figure 1) by taking the examples with normalised NGS counts<0.01 and a loss <1. These have a median plddt of 85.0, which is slightly higher than the median of all examples with normalised NGS counts<0.01 (83.6). Note that all plDDTs are quite high in this case, which is a characteristic of sequences designed with classical methods such as Rosetta. Designed proteins are more stable and therefore easier to predict in single sequence mode as can be seen for e.g. Top7 here: biorxiv.org/content/10.1101/2021.08.24.457549v1.full.pdf and here: https://www.biorxiv.org/content/10.1101/2022.12.13.520346v1.full.pdf.
