# Supplementary material for: Peptide binder design with inverse folding and protein structure prediction
Source: Commun Chem. 2023 Oct 25;6:229. doi: 10.1038/s42004-023-01029-7 (PMC10600234; doi:10.1038/s42004-023-01029-7)
Supplement: Supplementary file 3 — Description of Additional Supplementary Files [file 42004_2023_1029_MOESM3_ESM.pdf]

Description of Additional Supplementary Files

File name: Supplementary Data 1

Description: Data for Figure 1

File name: Supplementary Data 2

Description: Data for Figure 2

File name: Supplementary Data 3

Description: Data for Figure 4

File name: Supplementary Data 4

Description: Data for Figure 5

File name: Supplementary Data 5

Description: Data for Figure 6

File name: Supplementary Software

Description: All code required to reproduce this study are available from: https://gitlab.com/patrickbryant1/binder_design and a snapshot of the code used is uploaded as Supplementary Software. This is a pipeline for designing peptide binders- Binder design using a combination of [Foldseek](https://search.), [ESM-IF1](https://www.biorxiv.) and [AlphaFold](https://www.). Foldseek is available under [GNU GPL-3.0](https://www.gnu.org/). ESM-IF1 is available under the [MIT license](https://opensource.). AlphaFold2 is available under the [Apache License, Version 2.0](http://www.apache.org/). The AlphaFold2 parameters are made available under the terms of the [CC BY 4.0 license] and have not been modified. The binder design pipeline here is available under the same licenses as a derivative of these methods.
